# Patient satisfaction and its health provider-related determinants in primary health facilities in rural China

**DOI:** 10.1186/s12913-022-08349-9

**Published:** 2022-07-26

**Authors:** Qiufeng Gao, Meili Liu, Lanxi Peng, Yang Zhang, Yaojiang Shi, Dirk E. Teuwen, Hongmei Yi

**Affiliations:** 1 Center for Experimental Economics in Education, Shaanxi Normal University, Xi’an, China; 2 Ghent University Hospital, Department of Neurology, Ghent, Belgium; 3 China Center for Agricultural Policy, School of Advanced Agricultural Sciences, Peking University, Beijing, China; 4 Institute for Global Health and Development, Peking University, Beijing, China

**Keywords:** Patient satisfaction, Primary health facility, Standardized patient, Rural China

## Abstract

**Background:**

Patient satisfaction is an important outcome measure of health service and is one of the main reasons for the gradual deterioration of doctor–patient relationships in China. This study used the standardized patient (SP) method to explore patient satisfaction and its health provider-related determinants among primary health facilities in rural China.

**Methods:**

The dataset comprised 1138 clinic cases in 728 rural primary health facilities in 31 counties, spread across four provinces. Information regarding the consultation interaction between the unannounced SPs and primary physicians was recorded. Patient satisfaction was gathered from the feedback of SPs after the visit.

**Results:**

The overall average score of SP satisfaction with rural primary health facilities was only 13.65 (SD = 3.22) out of 20. The SP scores were found to be consistent with those of real patients. After controlling variances in patient population via the SP method, the regression analysis demonstrated that health provider-related factors, such as physician-level characteristics, consultation process, affordability, and convenience, have a significant correlation with patient satisfaction among primary physicians. Among factors relating to physician-level characteristics, affordability, convenience and the consultation process of the visit, the quality of the consultation process (e.g., consultation time, proactively providing necessary instructions and other crucial information) were found to be the prominent determinants.

**Conclusions:**

This study revealed the need to improve patient satisfaction in primary health facilities in rural China. To solve this issue, we recommend that policies to increase medical service quality be implemented in rural primary healthcare systems.

**Supplementary Information:**

The online version contains supplementary material available at 10.1186/s12913-022-08349-9.

## Background

Patient satisfaction is a critical indicator that is commonly used to evaluate health service outcomes [1]. It refers to patients’ assessment of the healthcare services they received. Such assessments can help understand the needs of patients and identify service factors that need improvement [2]. In China, evidence shows that low levels of patient satisfaction with medical services is one of the main reasons for the deterioration of doctor–patient relationships, resulting in lower utilization of medical services, especially in primary care systems [3, 4]. Primary care-oriented health systems likely result in more equitable and better health outcomes, since primary care is more cost-effective [5]. The perfect primary care system cannot get rid of high degrees of patient satisfaction and stable relationships between primary physicians and patients [6]. Thus, focusing on patient satisfaction with primary health facilities (PHFs) is beneficial for addressing doctor–patient conflicts and improving health reform in China [7].

Although studies have suggested that Chinese residents are less satisfied with the rural health system than with the urban health system, data on PHFs in rural China (i.e., township health centers and village clinics) remain limited [3, 8]. In China, PHFs are responsible for the majority of regular health services to residents; visits to PHFs accounted for 31.78% of the total number of clinical visits in 2019 [9]. Thus, how patients’ medical conditions and concerns are discussed and treated in rural PHFs is an important question, to which the answer is not clear. Existing research on patient satisfaction is mainly concentrated in urban areas of China; few studies have specifically focused on rural PHFs [10, 11]. One study found that patient satisfaction with township health centers in rural areas was lower than that in upper county-level hospitals [8]. An investigation of rural PHFs in 2010 suggested that treatment outcomes were strongly correlated with patient satisfaction [11]. More recent studies have revealed poor levels of healthcare quality among rural PHFs in China [12], possibly resulting in low levels of patient satisfaction. To fully understand patient satisfaction with PHFs in rural China, and factors related to it, more relevant research is needed.

This problem in not unique to China; indeed, numerous developing countries face challenges in improving patient satisfaction with rural health systems [13–16]. Identifying factors relating to healthcare providers and their services is key to understanding and promoting patient satisfaction with rural PHFs. Empirical studies have explored factors related to patient satisfaction from two aspects: patient-related characteristics (e.g., age, educational background, and health status) and healthcare provider-related determinants (e.g., institution and physician characteristics, consultation process, affordability, and convenience) [1]. Given their importance as influencing factors, exploring determinants related to primary physicians would help create effective strategies to improve patient satisfaction in rural China. As a result of relatively limited health resources available to them, rural residents may have different expectations; thus, determinants related to health providers in rural areas may be quite different from those found in urban regions [14]. Unfortunately, as previous literature has suggested, not controlling or adjusting for confounding factors (such as patient characteristics) may have created difficulties in detecting intrinsic determinants of patient satisfaction related to health providers [1]. However, to our knowledge, relevant empirical studies to detect provider-related factors in rural PHFs of developing countries are scarce.

In this study, we used the standardized patient (SP) methodology to assess health provider-related determinants of patient satisfaction. An SP is an actor who is trained to present cases of specific diseases in a standardized manner [17]. This method not only controls for bias as a result of differences in patient population, but also targets specific diseases, which ensures inter-rater reliability between SPs and enables comparisons across different providers [18]. Since SPs seek healthcare unannounced in practical settings, detailed information regarding medical visits in real-world situations can be gathered by recording the interactions between SPs and physicians. The SP method has been recommended as an effective method for examining clinical performance with an emphasis on client outcomes [19–22]. In particular, although SPs may judge physicians more critically than actual patients, studies have found that SPs provide authentic feedback about patient experience [23, 24] and the ranking scores of SPs have proven to be consistent with those of actual patients in empirical studies [18, 25]. In other words, satisfaction as determined by SPs can predict the patient satisfaction of real patients, to a certain extent [18].

From a large sample of 728 rural PHFs in four provinces located in different regions of China, our overall objective is to explore determinants related to health providers on patient satisfaction among PHFs in rural China. This study has three specific objectives. The first was to describe primary physicians’ characteristics and the medical services they provide from interactions between them and SPs. The second was to evaluate the levels of patient satisfaction as reported by SPs, with regard to rural PHFs. The third was to identify the influencing factors related to health providers on the level of SPs’ satisfaction with rural PHFs.

## Methods

### Sampling

This study consisted of two sets of data from rural areas located in four provinces in China. Dataset 1 comprised information gathered from Sichuan, Shaanxi, and Anhui provinces in 2015, and Dataset 2 comprised data collected from Yunnan Province in 2017. These four provinces are located in the eastern, central, and western parts of China, and have a high number of rural low-income counties. In the survey year, the per capita GDP of Sichuan, Shaanxi, Anhui, and Yunnan provinces were 36,775 yuan ($5489), 47,626 yuan ($7108), 35,997 yuan ($5373), and 34,221 yuan ($5555), respectively, which was far below the national average of 59,660 yuan ($8904) [26, 27]. However, the proportions of the rural population in these four provinces were 52.31, 46.08, 49.50, and 53.31%, respectively, much higher than the national average of 41.48% [26, 27].

**Fig. 1:**
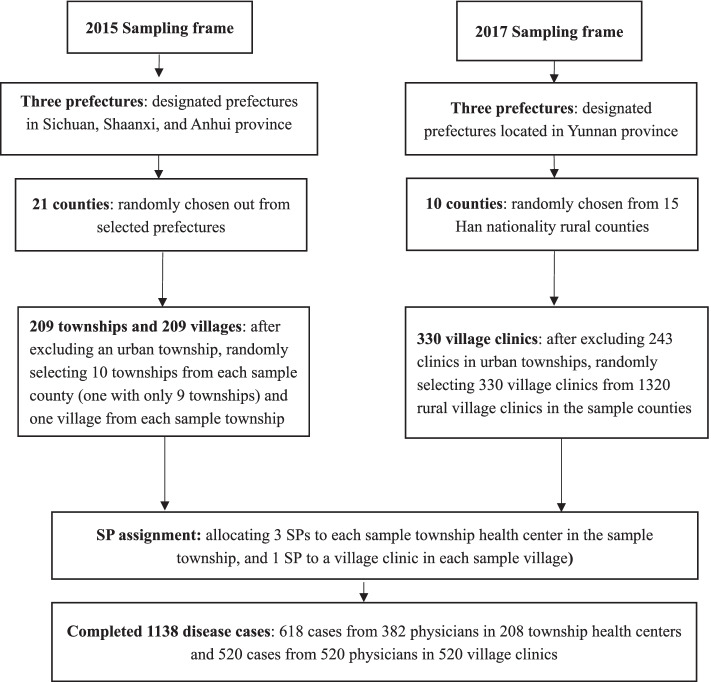
Flow chart of sample selection procedure

A multi-level random sampling method was adopted for both datasets. In Dataset 1, within three designated prefectures in the sample provinces, we randomly selected 21 of 24 rural counties. After excluding an urban township housing the county seat, we randomly selected ten townships in each sample county. Then one village was randomly selected within each sample township. Since there were only nine townships in one county, a total of 209 townships and 209 villages were included. Township health centers and village clinics in the selected townships and villages respectively, were identified as the study subjects. In Dataset 2, after excluding urban counties and ethnic minority autonomous regions from three prefectures, 10 counties were randomly selected from 15 Han nationality counties as the sample. After excluding a total of 243 clinics in urban townships in these counties, we randomly selected village clinics proportional to the number of village clinics in each sample county. In the end, a total of 330 village clinics were randomly selected from 1320 rural village clinics as the study subjects (Fig. 1).

### Standardized patients

We recruited local rural residents to act as SPs, which helped prevent them being identified as SPs by the physicians, since they represented actual patients commonly diagnosed by rural primary physicians. After training and screening, 78 SPs were selected to present four disease cases (viral diarrhea, unstable angina, tuberculosis, and asthma). These diseases are appropriate for the SP methodology as (1) these diseases are common in rural China; (2) there are no obvious physiological symptoms; (3) there is low risk that SPs would be exposed to invasive procedures or tests [28, 29]. All these SPs claimed that they have participated in the new rural cooperative medical system. In principle, we successively allocated three SPs randomly to one township health center, and one SP to one village clinic to report a case randomly determined beforehand. In the end, due to the absence of sample physicians during the SP visits, the unannounced SPs completed 1138 disease case presentations involving 618 cases from 382 physicians in 208 township health centers and 520 cases from 520 village clinicians in 520 village clinics (Fig. 1).

### Data collection

Data collection was carried out in two survey waves. In the first wave, we collected characteristics of the health facilities and the physicians via a structured questionnaire. Observable physician-level characteristics were included in the study. Specifically, in each sample PHF, we collected information regarding number of physicians, number of patients in the preceding week, and amount of equipment. Simultaneously, we enquired about the sex, age, education level, and medical qualification level of the physician.

In the second wave, we used the SP method to measure patient satisfaction and related factors from the interactions between the SPs and the physicians. After each consultation, the SP was debriefed with a structured questionnaire to evaluate their satisfaction with the physician. To measure the consultation process of the SP visit, we gathered information from variables, including duration of the consultation, whether the physician proactively provided diagnoses or medical advice, whether the SP was asked about basic information, and whether medical examinations (including examations performed by the SP during the visit, examinations suggested in higher-level facilities, and invasive examinations that were not completed but for which the SPs provided the results) or follow-up visits were suggested. Additionally, whether the consultation was interrupted by others was also recorded. In terms of the affordability and convenience, we asked about the number of patients waiting when the SP arrived, the duration of the waiting time, and the total cost.

### Patient satisfaction

This study adopted a structured questionnaire to evaluate SPs’ levels of patient satisfaction with primary physicians. The questionnaire contained four items: (1) “The physician made you feel relaxed and willing to describe symptoms and concerns to him/her”; (2) “The physician knew a lot about the disease”; (3) “In general, the physician gave you adequate explanations and instructions during the visit”; and (4) “The physician fully clarified and explained the treatment plans.” The responses were rated on a 5-point Likert-type scale, ranging from 1 = strongly disagree to 5 = strongly agree. The level of the SPs’ overall satisfaction was calculated from the summed scores of these four items.

To verify the robustness of the overall satisfaction scores, we first obtained SPs’ feedback regarding their subjective evaluations of the primary physicians. As the patient, we asked SPs whether they liked the physician they had visited and whether they would visit this physician again when they feel ill. Moreover, to test the robustness of our results from the perspective of real patients, local residents were asked to rate their satisfactions with the physicians in the selected village clinics. The satisfaction questionnaire for real patients comprised the following: (1) “Communication with the physician”; (2) “Disease diagnosis by the physician”; (3) “Inquiry and examination of your disease by the physician”; (4) “Treatment plans and medical advice provided by the physician.” All items were rated on a 10-point scale, with 1 indicating the lowest and 10 representing the highest level of satisfaction. The total scores of these four items represented the overall satisfaction level of real patients. Further, these rural residents also subjectively rated their satisfaction with medical service quality provided by the physicians on a scale of 1 to 10. In total, we gathered data from 632 randomly selected residents from 105 villages.

The SPs’ satisfaction questionnaire was tested on a sample of 1138 respondents. Cronbach’s alpha showed acceptable reliability (α = 0.76). Because the value was greater than 0.70, the scale and the data had good internal consistency. We also tested the structural validity of the questionnaire and found that the Kaiser-Meyer-Olkin (KMO) value was 0.739, and the Bartlett spheroid test value was 1209.78 (*p* < .001). The satisfaction questionnaire for real patients, tested on 632 rural residents, had the following values: Cronbach’s α = 0.93, KMO = 0.858, and Bartlett’s spheroid test = 1959.70 (*p* < .001). These results showed that the questionnaires had good reliability and validity to represent patient satisfaction.

### Statistical analysis

To explore associations between health provider-related factors and SPs’ satisfaction with rural PHFs, multiple linear regression models with fixed effects were used. The regression model is specified as:

1 $${\mathrm{y}}_{\mathrm{ij}}={\upalpha}_4+{\upbeta}_4\ast {\mathrm{F}}_{\mathrm{ij}1}+{\upbeta}_5\ast {\mathrm{F}}_{\mathrm{ij}2}+{\upbeta}_6\ast {\mathrm{F}}_{\mathrm{ij}3}+{\mathrm{r}}_{\mathrm{ij}}+{\upmu}_{\mathrm{ij}}+{\mathrm{v}}_{\mathrm{ij}}+{\mathrm{w}}_{\mathrm{ij}}+{\mathrm{z}}_{\mathrm{ij}}+{\upvarepsilon}_{\mathrm{ij}}$$

where y_ij_ represents the overall satisfaction score of a particular SP with physician j of sample case i. F_ij1_, F_ij2_, and F_ij3_ respectively, represent the characteristics of the facilities and the physicians, factors of affordability and convenience, and factors of the consultation process during the SP’s visit to physician j in sample case i. We included only one above factor at a time as the independent variable in the model knowing that these different factors are correlated. We simultaneously estimated a full model with all these three explanatory variables to predict patients’ satisfaction. r_ij_ indicates the SP’s fixed effect. μ_ij_ indicates the fixed effect of disease type. v_ij_ indicates the fixed effect of visiting day. w_ij_ and z_ij_ respectively indicate the facility level (township health center or village clinic) and the survey year (2015 or 2017). ε_ij_ is the error term.

To verify the robustness of the patient satisfaction levels of the SPs, we used multiple-regression analysis to evaluate the association between SP satisfaction and real patient satisfaction with the same physician. To adjust for systematic differences between SP raters within the same disease case, we considered the adjusted overall satisfaction scores of SPs (a predicted value of SP satisfaction score from formula (1)) in the model. The model is specified as:

2 $${\mathrm{Y}}_{\mathrm{j}}={\upalpha}_0+{\upalpha}_1\ast {\hat{\mathrm{y}}}_{\mathrm{j}}+{\upalpha}_2\ast {\mathrm{X}}_{\mathrm{j}}+{\mathrm{t}}_{\mathrm{j}}+{\upvarepsilon}_{\mathrm{j}}$$

where Y_j_ represents satisfaction outcome indicators of real patients (including the overall satisfaction score and the subjective score of medical service quality) with the physician j. ${\hat{\mathrm{y}}}_{\mathrm{j}}$ indicates the adjusted overall satisfaction score of the SP with physician j. X_j_ is a set of control variables of patients’ characteristics, such as sex, age, education level, leadership status, and health status. t_j_ indicates the village fixed effect, and ε_j_ is the error term.

## Results

### Characteristics of selected facilities and physicians

Table 1 presents the characteristics of the facilities and the physicians in the surveyed PHFs in rural China. According to the survey data, 3.6 physicians on average provided consultation services in each rural PHF. Approximately 102 patients in the preceding week were seen at a typical PHF during the survey period. The average quantity of equipment used for medical examinations across the PHFs was 16.2. Of the interviewed physicians, approximately four-fifths (77.9%) were men, and the average age was close to 45. More than three-fifths (62.1%) of the physicians did not complete academic college. Although all of these physicians were certified, only 27.5% of them held a “Practising Physician” certificate with a level above the “Rural Physician” and “Assistant Practising Physician” certificates. These characteristics among sample township health centers and village clinics are reported separately in Additional file 1.

**Table 1:** Characteristics of facilities and physicians in sample primary health facilities

Variable	Mean (SD)/n (%)
**Characteristics of facilities (*n*** **= 728)**	
Number of physicians	3.6 (4.7)
Number of patients in the preceding week	102.1 (276.5)
Amount of equipment	16.2 (4.0)
**Characteristics of physicians (*n*** **= 902)**	
Sex	
Male	703 (77.9)
Female	199 (22.1)
Age (years)	45.2 (10.5)
Education	
College or above	342 (37.9)
Below college	560 (62.1)
Qualification certificate	
Practising Physician	248 (27.5)
Assistant Practising Physician or Rural Physician	654 (72.5)

### Consultation process, affordability, and convenience of SP visits

Table 2 shows the consultation process of SP visits to the selected rural PHFs. The average consultation time across the 1138 cases was approximately 2.8 minutes. In all, less than one-third of the selected physicians took the initiative to inform the SPs of their diagnosis (25.4%) or volunteered medical advice for the represented disease (29.6%). Only 58.2% of the primary physicians requested basic information regarding the SP during the consultation. Physical examinations were recommended to 63.0% of the SPs and follow-up visits were recommended to 14.6%. More than one-third (34.7%) of the consultations between primary physicians and SPs were interrupted by other people.

**Table 2:** Consultation process, affordability and convenience of SP visits (*n* = 1138)

Variable	Mean (SD)/n (%)
**Consultation process**	
Duration of the consultation (minutes)	2.8 (3.2)
Proactively provided diagnoses	
Yes	289 (25.4)
No	849 (74.6)
Proactively provided medical advice	
Yes	337 (29.6)
No	801 (70.4)
Asked the basic information of the SP	
Yes	662 (58.2)
No	476 (41.8)
Suggested medical examinations	
Yes	717 (63.0)
No	421 (37.0)
Suggested for follow-up visits	
Yes	166 (14.6)
No	972 (85.4)
The consultation was interrupted by others	
Yes	395 (34.7)
No	743 (65.3)
**Affordability and convenience**	
Number of patients waiting when the SP arrived	0.9 (2.1)
Duration of the waiting time (minutes)	4.8 (9.1)
Medical cost (yuan)	15.1 (24.4)

Information regarding the affordability and convenience of the SP visits is also reported in Table 2. In terms of the waiting time, for these visits, only one patient on average was waiting in line to see a doctor; the average waiting time was 4.8 minutes. The average consultation fee charged by the selected physicians was 15.1 yuan ($2.3).

### Satisfaction of SPs with primary physicians

Table 3 describes the results of the SPs’ satisfaction with physicians in the selected PHFs. Regarding the four sub-items, the highest percentage of SP satisfaction was on physicians’ service attitude. Specifically, nearly 79% of SPs thought that the attitude of the primary physicians was gentle and thus, it relaxed them enough to discuss the disease conditions with the physicians. Meanwhile, 64.1% of the SPs expressed dissatisfaction with the physicians’ clinical knowledge, the domain they were least satisfied with. Overall, more than 40% of SPs expressed dissatisfaction with the physicians they visited, who did not give them sufficient explanations (45.7%) or fully clarify treatment plans (41.3%). The same situation also appeared in sample village clinics and township health centers respectively. Additionally, the score of overall satisfaction and three items of the four sub-items in sample village clinics were both slightly lower than those in sample township health centers (*p* < .001).

**Table 3:** SPs’ satisfaction with primary physicians in rural sample PHFs

Variables	Likert 5-Point Scale of Patient Satisfaction, n (%)					Satisfaction Score	T-test with Health facilities Level
	Strongly Disagree	Disagree	Neutral	Agree	Strongly Agree	*Mean ± SD*	*p-value*
**Full Samples(*n*** **= 1138)**							
The physician made you feel relaxed and willing to describe symptoms and concerns to him/her	30 (2.6)	63 (5.5)	149 (13.1)	664 (58.4)	232 (20.4)	3.88 ± 0.88	< 0.001
The physician knew a lot about the disease	96 (8.4)	170 (14.9)	464 (40.8)	323 (28.4)	85 (7.5)	3.12 ± 1.03	< 0.001
In general, the physician gave you adequate explanations and instructions during the visit	67 (5.9)	200 (17.6)	253 (22.2)	495 (43.5)	123 (10.8)	3.36 ± 1.07	< 0.001
The physician fully clarified and explained the treatment plans ^a^	60 (5.3)	174 (15.3)	236 (20.7)	512 (45.0)	118 (10.4)	3.30 ± 1.20	0.3630
Overall satisfaction score	*N/A*	*N/A*	*N/A*	*N/A*	*N/A*	13.65 ± 3.22	< 0.001
**Township health centers(*n*** **= 618)**							
The physician made you feel relaxed and willing to describe symptoms and concerns to him/her	15 (2.4)	32 (5.2)	34 (5.5)	375 (60.7)	162 (26.2)	4.03 ± 0.86	*N/A*
The physician knew a lot about the disease	53 (8.6)	88 (14.2)	220 (35.6)	191 (30.9)	66 (10.7)	3.21 ± 1.09	*N/A*
In general, the physician gave you adequate explanations and instructions during the visit	35 (5.7)	109 (17.6)	95 (15.4)	292 (47.2)	87 (14.1)	3.46 ± 1.11	*N/A*
The physician fully clarified and explained the treatment plans ^a^	37 (6.0)	94 (15.2)	65 (10.5)	308 (49.8)	81 (13.1)	3.33 ± 1.34	*N/A*
Overall satisfaction score	*N/A*	*N/A*	*N/A*	*N/A*	*N/A*	14.03 ± 3.40	*N/A*
**Village clinics(*n*** **= 520)**							
The physician made you feel relaxed and willing to describe symptoms and concerns to him/her	15 (2.9)	31 (5.9)	115 (22.1)	289 (55.6)	70 (13.5)	3.71 ± 0.88	*N/A*
The physician knew a lot about the disease	43 (8.3)	82 (15.8)	244 (46.9)	132 (25.4)	19 (3.6)	3.00 ± 0.94	*N/A*
In general, the physician gave you adequate explanations and instructions during the visit	32 (6.2)	91 (17.5)	158 (30.4)	203 (39.0)	36 (6.9)	3.23 ± 1.02	*N/A*
The physician fully clarified and explained the treatment plans ^a^	23 (4.4)	80 (15.4)	171 (32.9)	204 (39.2)	37 (7.1)	3.26 ± 1.01	*N/A*
Overall satisfaction score	*N/A*	*N/A*	*N/A*	*N/A*	*N/A*	13.21 ± 2.94	*N/A*

^a^ Since 38 physicians did not give any treatment plans, their score of this item was assigned to zero

**Fig. 2:**
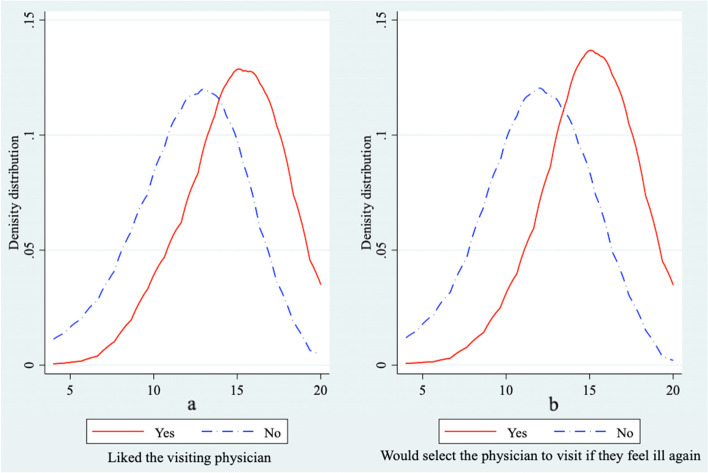
Distribution of overall satisfaction scores of SPs among different groups

In sum, the average overall score of SP satisfaction with primary physicians was 13.65 (SD = 3.22) out of a maximum of 20. Robustness checks of the overall satisfaction scores of the SPs are reported in Fig. 2. The physicians whom the SPs liked and would select to visit again were those that received higher scores of SP satisfaction, indicating that the overall SP satisfaction scores represent a definite degree of patient satisfaction. In addition, the adjusted satisfaction scores of the SPs were found to be significantly correlated with the overall satisfaction scores of real patients (Table 4, *p* < .01) and the subjective scores of real patients on medical service (*p* < .05). This finding implied that SP satisfaction scores regarding primary physicians could consistently predict those of real patient satisfaction.

**Table 4:** Multiple regression analyses between SP satisfaction and real patient satisfaction (*n* = 632)

Variable	(1)		(2)	
	Overall satisfaction score of real patients		Subjective score of real patients on medical service	
	B (SE)	*P* value	B (SE)	*P* value
Adjusted satisfaction score of SPs	7.73 (2.41)	0.001**	1.45 (0.69)	0.035*

In Dataset 2, we randomly selected villages and local residents within the villages to conduct patient satisfaction questionnaire survey. In total, 632 residents from 105 villages were included in the study. In the regression analysis, we also controlled village fixed effect and characteristics of rural residents, such as sex, age, education level, leadership status, and health status

### Health provider-related determinants of patient satisfaction

We further identified health provider-related determinants of the SPs’ satisfaction, including the characteristics of the facilities and the physicians, affordability and convenience, and the consultation process. Multiple regression analysis results are presented in Table 5. First, among all the observable variables of basic characteristics, only the age of physicians was significantly correlated with the SPs’ satisfaction. Specifically, younger physicians that provided medical service were more likely to satisfy the SPs (*p* < .01). Characteristics of the PHFs and other observable physician-related variables did not have significant relations with SP satisfaction. Second, in terms of affordability and convenience, longer waiting times were positively correlated with higher scores of SP satisfaction (*p* < .05). However, there were no significant results regarding the number of waiting patients or medical costs.

**Table 5:** Multiple linear regression analyses on health provider-related determinants of patient satisfaction (*n* = 1138)

Variable	Overall satisfaction score of SPs							
	(1)		(2)		(3)		(4)	
	B (SE)	*P* value	B (SE)	*P* value	B(SE)	*P* value	B(SE)	*P* value
**Characteristics of facilities and physicians**								
Number of physicians^a^								
1 > =3.6	−0.06 (0.32)	0.847					−0.13 (0.29)	0.645
0 < 3.6	0.00						0.00	
Number of patients in the preceding week ^a^								
1 > =102.1	0.24 (0.22)	0.275					0.19 (0.20)	0.346
0 < 102.1	0.00						0.00	
Amount of equipment ^a^								
1 > =16.2	0.09 (0.25)	0.734					0.04 (0.23)	0.870
0 < 16.2	0.00						0.00	
Sex								
1 = Male	−0.11 (0.24)	0.648					−0.18 (0.23)	0.414
0 = Female	0.00						0.00	
Age ^a^								
1 > =45.2	−0.61 (0.21)	0.004**					−0.43 (0.20)	0.030*
0 < 45.2	0.00						0.00	
Education								
1 = Collage or above	0.20 (0.23)	0.399					0.24 (0.22)	0.269
0 = Below Collage	0.00						0.00	
Qualification certificate								
1 = Practising Physician	−0.52 (0.27)	0.052					−0.48 (0.25)	0.052
0 = Assistant Practising Physician or Rural Physician	0.00						0.00	
**Affordability and convenience**								
Number of patients waiting when the SP arrived ^a^								
1 > =0.9			−0.04 (0.20)	0.837			−0.07 (0.20)	0.716
0 < 0.9			0.00				0.00	
Duration of the waiting time ^a^								
1 > =4.8			0.44 (0.23)	0.053			0.49 (0.22)	0.027*
0 < 4.8			0.00				0.00	
Medical cost ^a^								
1 > =15.1			0.15 (0.21)	0.473			−0.10 (0.19)	0.614
0 < 15.1			0.00				0.00	
**Consultation process**								
Duration of the consultation ^a^								
1 > =2.8					0.90 (0.21)	< 0.001***	0.89 (0.21)	< 0.001***
0 < 2.8					0.00		0.00	
Proactively provided diagnoses								
1 = Yes					1.06 (0.22)	< 0.001***	1.07 (0.22)	< 0.001***
0 = No					0.00		0.00	
Proactively provided medical advice								
1 = Yes					1.17 (0.21)	< 0.001***	1.15 (0.21)	< 0.001***
0 = No					0.00		0.00	
Asked the basic information of the SP								
1 = Yes					0.25 (0.19)	0.194	0.30 (0.19)	0.108
0 = No					0		0	
Suggested medical examinations								
1 = Yes					1.22 (0.29)	< 0.001***	1.17 (0.28)	< 0.001***
0 = No					0.00		0.00	
Suggested for follow-up visits								
1 = Yes					0.26 (0.27)	0.349	0.17 (0.28)	0.544
0 = No					0.00		0.00	
The consultation was interrupted by others								
1 = Yes					0.14 (0.18)	0.423	0.10 (0.18)	0.601
0 = No					0.00		0.00	

We also controlled fixed effects of SPs, types of diseases and visiting day, facility level and survey year in the regression analysis

^a^The dummy variables were generated according to the mean value

Third, regarding the consultation process determinants, the duration of consultations, whether the physicians proactively gave diagnoses or advice, and whether the SPs were given suggestions regarding examinations, were significantly correlated with the SPs’ level of satisfaction. Specifically, longer lasting consultations resulted in higher satisfaction levels (*p* < .001). In addition, SPs were more satisfied with physicians who actively diagnosed or provided medical advice during the visit (*p* < .001). SPs were also more likely to be satisfied with the primary physicians if the consulted physicians recommended medical examinations (*p* < .001). Except for the waiting time variable, the results consistently reported significant determinants when all the health provider-related factors were combined in the multiple regression analysis.

## Discussion

This study is the first to use the SP method to explore patient satisfaction and its health provider-related determinants among rural PHFs in China. In the study, the robustness checks were not only consistent between SP satisfaction scores and their subjective evaluation results as the patient, but also revealed significantly positive correlations between SP satisfaction scores and real patient satisfaction scores, after controlling for patient characteristics. These findings certify that SP satisfaction can control variances in patient populations and reflect the patient satisfaction of real patients [18]. Accordingly, we evaluated health provider-related factors of patient satisfaction with rural PHFs using a large sample of 1138 disease cases presented by SPs to physicians in 728 rural PHFs located in four regions of China. Our main findings based on the SP method are presented below.

First, the survey data indicated that rural PHFs have limited human resources, and the quality of their care services was poor. In line with previous studies, we found that physicians’ educational background and clinical qualifications in rural PHFs were inferior [12, 30]. In our sample, slightly more than one-third (37.9%) of primary physicians had a college degree and around three-quarters (72.5%) did not obtain practising physician certifications. The selected physicians provided SPs with short visits, averaging 2.8 minutes. Similarly, a comparative analysis of primary doctors in 67 countries found that the average consultation time of Chinese physicians was relatively short and ranked in the bottom position [31]. The short consulting period resulted in the physicians not explaining medical information clearly to rural patients. Less than one-third of the selected physicians proactively informed SPs of their diagnostic results (25.4%) and provided medical advice (29.6%). If patients do not correctly understand and treat their health problems, it might be difficult to ensure their compliance to the treatment and return to the primary health care [2]. Moreover, as some studies have shown, the low quality of medical services may be the main factor for the underutilization of services in PHFs in rural China [32–34]. The small number of patients waiting in line and the limited waiting time during SP visits confirmed this issue.

Second, our findings show that approximately half of the consultation services provided by the physicians were in some way unsatisfactory, which was twice as high as that reported in surveys of upper-level hospitals in both rural and urban areas [3, 35, 36]. Our findings were also similar to those conducted in other developing countries, such as Ethiopia and Ghana [13, 16]. The low scores of patient satisfaction in this study are similar to previous findings conducted among primary physicians of rural China [8, 10]. More specifically, the average satisfaction score of SPs with the selected PHFs (3.41 out 5 per item) was close to that of real patients conducted in rural township health centers (3.67 out 5 per item) [8]. Additionally, in line with other studies, we found that patients were most satisfied with the attitude of the primary physicians and least satisfied with their service quality [8, 37, 38]. The data showed that only 35.85% of the SPs were satisfied with the primary physicians’ clinical performance. The low percentage of satisfaction with medical service quality was also lower than that reported by previous findings, in which around half the patients expressed satisfaction with upper-level hospitals [8, 36].

Third, as previous findings have suggested, the regression analysis demonstrated that provider-related factors were determinants of patient satisfaction with rural PHFs [1]. Specifically, in the observable physician-level characteristics, the age of physicians was found to be the only significant factor of SP satisfaction with primary physicians. This may be because younger physicians show more enthusiasm for healthcare and possess better communication skills, which may be lacking in older doctors [39, 40]. For example, younger physicians prefer to use explanations and communication skills rather than drugs to solve patients’ problems [39], which are among the most effective determinants of patient satisfaction [11]. With regard to affordability and convenience of medical visits, longer waiting times were positively correlated with higher levels of patient satisfaction. Although some studies have suggested that longer waiting time indicated less accessibility and reduced patient satisfaction [41, 42], the positive correlation results may be due to the low number of patients waiting in rural PHFs. A certain waiting time means that local residents did actually seek medical treatment from the physician, which helps promote SPs’ favorable impression and trust in the physician.

Additionally, the regression results suggested that, compared with other provider-related characteristics, factors related to the consultation process played a more important role in patient satisfaction with rural PHFs [11, 35]. In terms of consultation process determinants, consistent with previous studies, we found that longer consultation time resulted in higher patient satisfaction [43–45], as it was more likely to result in an increase in the amount of information given to patients [43, 45]. Our findings verify that physicians who proactively provided necessary instructions and other crucial information (diagnostic results, medical checking or advice) received higher patient satisfaction scores. Consistent with our research, a series of studies have found that being given sufficient instructions and explanations are critical in improving patient satisfaction [46–48]. As we discussed above, the appropriate duration of consultation, providing diagnostic results, and medical advice were vital for the quality of treatment outcomes [43, 45, 46, 48]. To correctly manage the disease cases included in the study, medical screening tests are also needed to make careful judgements. This means whether the services adhere to standards of clinical diagnoses and treatment plays a significant role in improving patient satisfaction. As previous studies suggested, our findings confirmed that important health provider-related factors that influence patient satisfaction were the health service quality indicators [1, 49]. Specially, in primary care settings, the behavior of physicians, including consultation quality, have an immediate impact on patients’ medical concerns and health recovery, and thus have been recognized as the main factor of patient satisfaction [10, 50]. However, as previous findings suggested, our results consistently found that primary physicians cannot provide high-quality medical service that meets patients’ expectations [37, 51, 52], which could result in the deterioration of patient satisfaction with PHFs. Some studies have revealed that the low levels of patient satisfaction have partly lead to the low utilization of rural PHFs [3, 4]. This will result in more cost-effective primary medical services that can not deliver fairer and better health outcomes effectively [5], nor helping to drive healthcare reform in China [7]. Therefore, training primary physicians to improve service quality is imperative from the perspective of improving patient satisfaction in rural China [11, 53, 54].

There are several limitations in this study. First, since the SPs represented only certain types of diseases, the data of this study cannot analyze patient satisfaction with regard to actual patients with different disease conditions. Second, since this was a cross-sectional study, causal relations could not be identified. Our results are concentrated on correlation analysis. Third, this study only included rural areas in four provinces in China, and may not represent patient satisfaction with rural PHFs across the country.

## Conclusions

In conclusion, this study revealed low levels of patient satisfaction with PHFs in rural China. Health provider-related determinants play a role in variations in patient satisfaction; poor quality of the consultation process provided by primary physicians was recognized as the dominant factor resulting in lower satisfaction. Our findings suggest the urgency of improving patient satisfaction in China’s rural PHFs. Enhancing the service quality of rural primary physicians is an effective solution to this problem. Therefore, the Chinese government should pay more attention to the medical services provided by rural PHFs, and provide training to improve medical services.

## Supplementary Information

**Additional file 1.** Characteristics of facilities and physicians in sample township health centers and village clinics

## Data Availability

Public availability of data would compromise privacy of participants. Data will be made available from the corresponding author on reasonable request.

## Abbreviations

PHFs: Primary health facilities
SP: Standardized patient
