# Supplementary material for: Patient satisfaction and its health provider-related determinants in primary health facilities in rural China
Source: BMC Health Serv Res. 2022 Jul 26;22:946. doi: 10.1186/s12913-022-08349-9 (PMC9316702; doi:10.1186/s12913-022-08349-9)
Supplement: Supplementary file 1 — Additional file 1. Characteristics of facilities and physicians in sample township health centers and village clinics [file 12913_2022_8349_MOESM1_ESM.docx]

**Additional file 1. Characteristics of facilities and physicians in sample township health centers and village clinics**

| Variable | Mean (SD)/n (%) | |
| --- | --- | --- |
|  | THC（N=208） | VC（N=520） |
| **Characteristics of facilities** |  |  |
| Number of physicians | 7.7 (7.2) | 1.9 (1.0) |
| Number of patients in the preceding week | 110.2 (98.0) | 98.8 (321.3) |
| Amount of equipment | 15.2 (3.0) | 16.6 (4.2) |
|  | THC（N=382） | VC（N=520） |
| **Characteristics of physicians** |  |  |
| Sex |  |  |
| Male | 328 (85.86) | 375 (72.12) |
| Female | 54 (14.14) | 145 (27.88) |
| Age (years) | 43.2 (10.0) | 46.6 (10.6) |
| Education |  |  |
| College or above | 238 (62.30) | 104 (20.00) |
| Below college | 144 (37.70) | 416 (80.00) |
| Qualification certificate |  |  |
| Practising Physician | 234 (61.26) | 14 (2.69) |
| Assistant Practising Physician or Rural Physician | 148 (38.74) | 506 (97.31) |

Note：THC refers to township health centers and VC refers to village clinics.
